# Vitamin D improves endothelial dysfunction and restores myeloid angiogenic cell function via reduced CXCL-10 expression in systemic lupus erythematosus

**DOI:** 10.1038/srep22341

**Published:** 2016-03-01

**Authors:** John A. Reynolds, Sahena Haque, Kate Williamson, David W. Ray, M. Yvonne Alexander, Ian N. Bruce

**Affiliations:** 1 Arthritis Research UK Centre for Epidemiology, Centre for Musculoskeletal Research, Institute of Inflammation and Repair, Manchester Academic Health Science Centre, University of Manchester, Manchester, UK; 2 Institute of Cardiovascular Science, Manchester Academic Health Science Centre, University of Manchester, Manchester, UK; 3 Rheumatology Department, University Hospital of South Manchester NHS Foundation Trust, Manchester, UK; 4 Department of Musculoskeletal Biology, Faculty of Health & Life Sciences, University of Liverpool, Liverpool, UK; 5 Endocrine Sciences Research Group, Institute of Human Development, Manchester Academic Health Science Centre, University of Manchester, Manchester, UK; 6 School of Healthcare Science, Manchester Metropolitan University, Manchester, UK; 7 NIHR Manchester Musculoskeletal Biomedical Research Unit, Central Manchester Foundation Trust, Manchester Academic Health Science Centre, Manchester, UK

## Abstract

Patients with systemic lupus erythematosus (SLE) have accelerated cardiovascular disease and dysfunctional endothelial repair mechanisms. Myeloid angiogenic cells (MACs), derived from circulating monocytes, augment vascular repair by paracrine secretion of pro-angiogenic factors. We observed that SLE MACs are dysfunctional and secrete pro-inflammatory cytokines. We also found that the vitamin D receptor was transiently expressed during MAC differentiation and that *in vitro*, calcitriol increased differentiation of monocytes into MACs in both SLE and in a model using the prototypic SLE cytokine, interferon-alpha. The active form of vitamin D (calcitriol) restored the SLE MAC phenotype towards that of healthy subjects with reduced IL-6 secretion, and normalised surface marker expression. Calcitriol also augmented the angiogenic capacity of MACs via the down-regulation of CXCL-10. In SLE patients treated with cholecalciferol for 12 weeks, the improvement in endothelial function correlated with increase in serum 25(OH)D concentrations independently of disease activity. We also show that MACs were able to positively modulate eNOS expression in human endothelial cells *in vitro,* an effect further enhanced by calcitriol treatment of SLE MACs. The results demonstrate that vitamin D can positively modify endothelial repair mechanisms and thus endothelial function in a population with significant cardiovascular risk.

Systemic lupus erythematosus (SLE) is an autoimmune, systemic, inflammatory disorder predominantly affecting women. Patients with SLE have a significantly increased risk of cardiovascular disease which is not explained by traditional risk factors^1^. Endothelial dysfunction and the loss of endothelial integrity precede the development of atherosclerosis^2^ and endothelial dysfunction, assessed by flow-mediated dilatation (FMD), is an established risk factor for future cardiovascular events in the general population^3^.

Circulating cells, including myeloid angiogenic cells (MACs), contribute to vascular homeostasis via the paracrine secretion of potent angiogenic factors^4^. In animal models, inhibition of MAC function is associated with reduced re-endothelialisation following carotid artery injury^5^. In addition, infusion of peripheral blood-derived MACs contribute directly to vascular repair post-myocardial infarction^6^. *In vitro* models of important MAC functions such as migration and angiogenesis, offer an opportunity to investigate novel vasculoprotective agents, including vitamin D. In the context of SLE, MACs are dysfunctional due, in part, to effects of interferon-alpha (IFNα)^7^,^8^. Furthermore, activated IFNα pathways are associated with both active disease and endothelial dysfunction in SLE patients^9^, supporting the use of IFNα treatment of MACs as a useful model of SLE.

Epidemiological studies show that vitamin D deficiency is associated with future cardiovascular events in the general population^10^ and correlates with the presence of endothelial dysfunction^11^ and carotid intima-medial thickness^12^. Of note, LDLR^−/−^VDR^−/−^ mice develop increased atherosclerosis independent of raised serum cholesterol levels^13^. Vitamin D deficiency is highly prevalent in SLE, due in part to the photosensitive nature of the disease^14^,^15^,^16^ and we have previously established that in SLE patients, arterial stiffness is associated with vitamin D status^17^.

High dose cholecalficerol supplementation has been shown to improve endothelial function over a short time course in some populations^18^,^19^,^20^,^21^, however the mechanism underpinning the improvements in endothelial function by vitamin D in SLE is unknown.

*In vitro* studies do not clearly support a direct positive effect of the active form of vitamin D (calcitriol) on mature endothelial cells^22^,^23^, however, in contrast, myeloid cell function has been shown to be modulated by calcitriol treatment *in vitro*^24^, suggesting that MAC responses may also be targeted by vitamin D. Recently Wong *et al.* (2014) demonstrated that the number of circulating CD45^−^CD117^+^Sca1^+^Flk1^+^cells (a putative precursor of MACs) was increased in 6 healthy subjects following treatment with 4,000 IU/day cholecalciferol. Furthermore, in 2 models of murine vessel wall injury, calcitriol promoted endothelial repair and angiogenesis; a phenomenon mediated via increased SDF-1 expression in the damaged vessel and increased MAC chemotaxis^25^.

This study describes the use of *in vitro* models of MAC function and the effectiveness of calcitriol to restore SLE MACs phenotype, augment the angiogenic capacity of MACs and to positively modulate the paracrine regulation of endothelial nitric oxide synthase (eNOS). The clinical relevance of these observations is investigated in terms of improvements in endothelial function in cholecalciferol-treated SLE patients.

## Results

### MACs from SLE patients are more numerous but show impaired function

**Figure 1:**
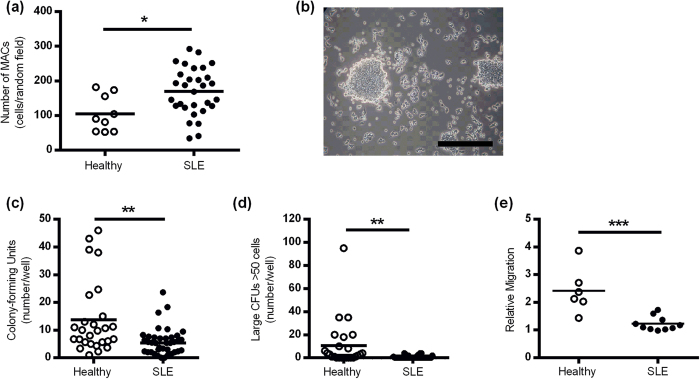
SLE MACs are dysfunctional *in vitro*. (**a**) Patients with SLE have an increased number of MACs after 7 days compared to HCs (n = 9 controls and n = 30 SLE patients). (**b**) Representative image of a CFU from a healthy subject. Scale bar =100 μm. The number of CFUs (**c**) and large colonies (>50 cells) (**d**) is significantly lower in SLE patients compared to HCs (n = 27 controls and n = 38 SLE patients). (**e**) The migratory capacity of SLE MACs towards SDF-1α is impaired in SLE. The graph shows the relative chemotaxis to SDF-1α compared to chemokinesis in n = 6 healthy subjects and n = 10 controls. Data show the mean number of MACs (over 3 random field per subject) or CFUs). *P < 0.05, **P < 0.01, ***P < 0.001 by Mann-Whitney U-test (**a,c**) and (**d**), and two-tailed t-test (**e**).

MACs were isolated from blood samples taken from SLE patients and healthy controls (HCs) and cultured for 7 days. The yield of MACs was significantly higher from SLE patients compared to healthy controls (Fig. 1a). The colony-forming unit (CFU) capacity of MACs was investigated as a global measure of MAC function. CFUs were studied in an additional cohort of n = 38 SLE patients and n = 27 HCs none of whom received cholecalciferol. The median (IQR) age was 53 (46, 58) and 43 (28, 55) years respectively Compared to healthy control MACs, we observed an impaired capacity of SLE MACs to generate colonies in terms of both number (median number 5.4 vs 9.8 per field, p = 0.0017, Fig. 1c) and size where the colonies formed were smaller using SLE cells (median number of colonies >50 cells per colony 0.0 vs. 3.0 per field, p = 0.0002). This difference in CFU number remained significant after adjustment for age (β [SE] 0.40 [2.49], p = 0.002). The migratory capacity of SLE MACs towards SDF-1α was also significantly impaired when compared to healthy controls (Fig. 1d). On further analysis there was no difference in migration between MACs from 25(OH)D deficient or replete SLE patients (not shown). These results demonstrate that whilst MAC number may be increased in this SLE population, the function is globally impaired.

### Calcitriol increases the differentiation of myeloid progenitor cells into MACs

**Figure 2:**
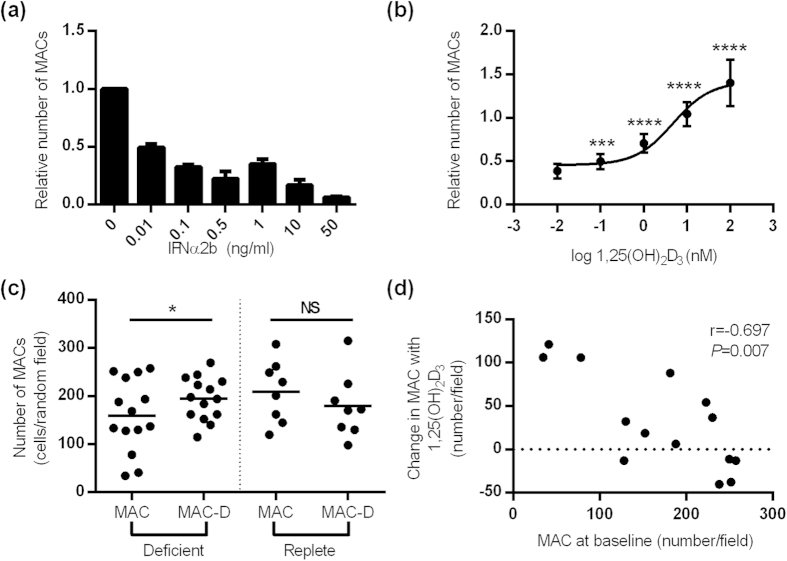
Calcitriol increases MAC number in SLE and in an IFN-model. (**a**) IFNα2b decreases the number of HC MACs in a dose-dependent manner (pooled from n = 5 healthy controls). (**b**) In a model of HC MACs treated with 0.1 ng/ml IFNα2b, calcitriol increases MAC number in a dose-dependent manner (n = 5 healthy subjects). The EC_50_ for this effect is 4.8 nM. (**c**) 10 nM calcitriol increases the number of MACs from vitamin D deficient but not vitamin D replete SLE patients (paired samples of n = 14 deficient patients and n = 8 replete). (**d**) The change in SLE MAC number (deficient patients) in response to calcitriol is negatively correlated with the number of untreated MACs (n = 14 patients). (**a**,**b**) show the mean ( ± SE) relative number of MACs compared to vehicle control. (**c**) shows the mean number of MAC and the bar shows mean for the group. (**d**) shows the Spearman correlation coefficient. *P < 0.05, ***P < 0.001, ****P < 0.0001 by paired t-test.

We isolated healthy MACs and incubated them in the presence of IFNα2b to determine whether this could replicate the SLE phenotype *in vitro*. Of note, a dose-response to IFNα2b (0.01–50 ng/ml) showed a marked relative reduction in the number of MACs present at day 8 (Fig. 2a), therefore, a low dose of IFNα2b (0.01 ng/ml) was used for further studies to retain cell viability. IFNα2b (0.01 ng/ml) treatment had no effect on any of the following parameters; i) the migratory capacity of MACs, ii) the adhesion of MACs to activated endothelium, or iii) the angiogenic capacity of MACs (data not shown), suggesting the abnormal phenotype of SLE MACs cannot be attributed to exposure of MACs to interferon-alpha in isolation.

However, the reduced survival of HC MACs in the presence of IFNα2b *in vitro* offered a model to investigate the direct effects of calcitriol, without concerns regarding the heterogeneity of SLE patients. In this model, calcitriol increased MAC number in a dose-responsive manner (Fig. 2b). This effect was significant at 1 nM concentration with an EC_50_ of 4.8 nM.

In vitamin D deficient (but not replete) SLE patients, culture with 10 nM calcitriol enhanced the expansion of MACs; the number of MACs (per random field) in deficient patients increased from mean (sd) 159.1 (75.3) to 194.5 (44.6) (p = 0.044) (Fig. 2c). The increased cell numbers in response to calcitriol was inversely related to the number of MACs in the absence of calcitriol (r = −0.697, p = 0.007), demonstrating that the increase in MACs in response to calcitriol is limited by a threshold effect (Fig. 2d).

### Calcitriol restores MAC phenotype in SLE

**Figure 3:**
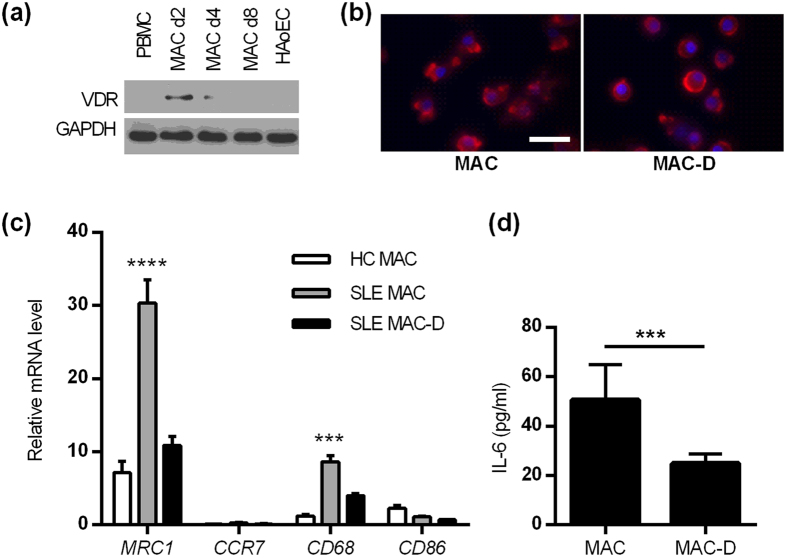
Calcitriol modulates MAC phenotype towards reduced differentiation. (**a**) Immunoblotting of the VDR shows increased but transient expression in MACs compared to PBMCs and HAoECs. (**b**) Calcitriol changes the morphology of SLE MACs. Image shows LDL-uptake by cells (red). Scale bar =50 μm. (**c**) qRT-PCR analysis shows increased expression of surface markers in SLE MACs which return towards HC levels of expression when cultured with calcitriol (n = 4 healthy and n = 5 SLE subjects). (**d**) Calcitriol reduces secretion of the pro-inflammatory cytokine IL-6 by SLE MACs (n = 10 subjects). Data in (**c**,**d**) show the mean (±SE). **P < 0.01, ***P < 0.001, ****P < 0.0001 by 2-way ANOVA (**c**) and paired t-test (**c**).

Expression of the VDR in PBMCs was similar to that detected in HAoECS (Fig. 3a). VDR expression markedly increased in MACs by day 2 (15.6-fold), declined by day 4 (5.1-fold from baseline), and returned to baseline (PBMC) levels by day 8. The culture of SLE MACs with calcitriol resulted in a morphological change in appearance, from the typical “fried egg” morphology of macrophages to a more rounded phenotype (Fig. 3b). In order to determine whether the architectural changes observed in the cells represent phenotypic shift in the MACs, we performed a gene ontology analysis of the dataset produced by Kupfer *et al.* (2013), comparing gene expression in healthy PBMCs in response to 10 nM calcitriol for 24 hours (see supplement for details)^26^. There was reduced expression of genes with roles in “haematological system development and function”. Within this group, there was significant down regulation of genes associated with phagocyte activation, movement, chemotaxis and inflammatory response.

These data were verified using qRT-PCR and compared to HC MACs, SLE MACs showed significantly increased expression of the general macrophage marker CD68, and the M2-specfic marker MRC1 (CD206) compared to the HC counterpart. When SLE MACs were incubated in the presence of calcitriol, expression of these markers returned towards the more normal phenotype observed in HC MACs (Fig. 3c).

Furthermore in the healthy PBMC dataset from Kupfer *et al.*, calcitriol down-regulated the expression of a number of genes related to macrophage differentiation including IL-6, IL-15, CD40 and MMP-9. To validate this data, using IL-6 as an exemplar, we measured the secretion of IL-6 by SLE MACs in response to calcitriol using ELISA. We found that IL-6 secretion by clacitriol-treated SLE MACs was significantly lower compared to untreated cells (24.9[11.8] vs. 50.7[45.0]pg/ml, p = 0.0002) (Fig. 3d). These data suggest that calcitriol may restore the activation status of SLE macrophages, towards that of HC, resulting in lower expression of pro-inflammatory cytokines.

### Angiogenic capacity of MACs is increased by calcitriol via down-regulation of CXCL-10

**Figure 4:**
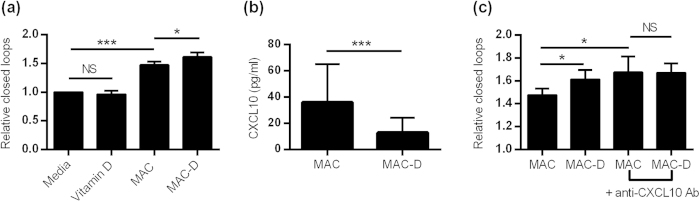
Calcitriol increases the angiogenic capacity of SLE MACs but not MAC migration or adhesion. (**a**) Angiogenic capacity of MACs in the Matrigel model is increased by isolating MACs with calcitriol whilst calcitriol alone is not pro-angiogenic (n = 8 patients). (**b**) Calcitriol reduces the expression of CXCL-10 by SLE MACs (n = 10 patients). (**c**) Blockade of CXCL-10 increases the angiogenic capacity of SLE MACs and attenuates the effect of culture with calcitriol (n = 3 experiments). Columns show means (±SE). *P < 0.05, ***P < 0.001 by ratio paired t-tests where indicated.

We confirmed that HC MACs have a pro-angiogenic capacity *in vitro* using a Matrigel angiogenesis model (see supplementary data, Fig. S1). The conditioned media from SLE MACs expanded in the presence of calcitriol (MAC-D) had significantly augmented angiogenic capacity (Fig. 4a). This was not due to the presence of calcitriol in the conditioned media, as calcitriol alone did not have an effect on endothelial network formation. There was no association between the change in MAC number and the increase in angiogenic capacity, suggesting that calcitriol may modify the MAC secretome in order to promote angiogenesis. Bioinformatics analysis of PBMC response to calcitriol (as described above) demonstrated up-regulation of 8 pro-angiogenic molecules, and down-regulation of 8 anti-angiogenic molecules (see supplementary data tables S2 and S3).

We postulated that CXCL-10 (IP-10) may be important, as this cytokine was increased in the serum of SLE patients and has anti-angiogenic functions in an *in vitro* Human Umbilical Vein Endothelial Cell (HUVEC) model^27^. In keeping with the data above showing reduced expression of IL6 in response to calcitriol, we found that MACs incubated in the presence of calcitriol (MAC-D), secreted lower concentrations of CXCL-10 (36.0 [91.9] vs. 12.9[36.0]pg/ml, p = 0.0006) (Fig. 4b). An anti-CXCL-10 neutralising antibody was then used to block the anti-angiogenic effects of CXCL-10 produced by SLE MACs in the angiogenesis model. In the angiogenesis assay, anti-CXCL-10 antibodies attenuated the pro-angiogenic effect of vitamin D (Fig. 4c). In other models of MAC function, SLE MACs expanded in the presence of calcitriol (MAC-D) showed no difference in migration or adhesion (not shown). These results demonstrate that calcitriol increases the angiogenic capacity of MACs by the down-regulation of CXCL-10.

### Vitamin D-treated SLE MACs modulate eNOS expression

**Figure 5:**
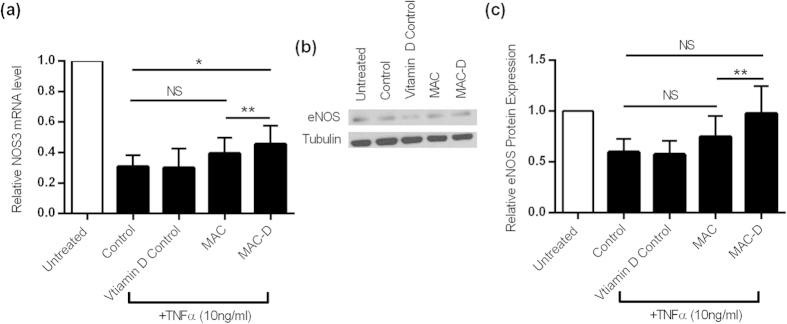
MAC regulation of endothelial cell eNOS expression. (**a**) TNFα down-regulates eNOS transcript expression in HAoECs. Calcitriol increased the ability of MAC-conditioned media (MAC-D) to partially inhibit this down-regulation whilst MACs cultured without vitamin D had no significant effect (MAC). (**b**) Representative immunoblot of the change in eNOS expression in HAoEC. (**c**) eNOS protein expression shows the same pattern as the transcript with greatest effect with vitamin D-treated MACs but no effect of calcitriol in isolation. (**a**,**c**) show results from n = 3 and n = 4 patients respectively. *P < 0.05, **P < 0.01 by ratio paired t tests.

The conditioned media from SLE MACs had no effect on eNOS expression in resting HAoECs (not shown). Tumour necrosis factor-alpha (TNFα) was therefore used to activate the endothelial cells and down-regulate eNOS. In the presence of TNFα, MAC media had a small protective effect on NOS3 expression which was further increased in the presence of MAC-D media (Fig. 5a). There was a good correlation of expression at the protein level, where MAC-D enhanced eNOS protein expression compared to SLE MACs (Fig. 5b,c). Calcitriol alone had no effect on NOS3 expression (transcript or protein level).

In summary, the *in vitro* data presented, demonstrate that calcitriol modifies the phenotype of SLE MACs, restoring them towards those of healthy controls, and augments both the angiogenic capacity of MACs and the paracrine regulation of eNOS.

### Vitamin D improves endothelial function in SLE patients

In our clinical cohort we compared vitamin D deficient (25(OH)D < 20 ng/ml) to vitamin D replete (25(OH)D > 30 ng/ml) patients. Vitamin D deficient patients were younger (median [IQR] age 46.9 [44.5, 57.6] vs. 57.8 [52.7, 64.6] years respectively, p = 0.007), were more likely to have a history of renal disease (7/22 [71.8%] vs. 1/18 [5.6%], p = 0.039) and to be taking corticosteroids or other immunosuppressive therapies (13/22 [59.1%] vs. 3/18 [16.7%], p = 0.006) (Table 1). Systolic blood pressure was higher in the deficient group (median [IQR] 124 [120,135] vs. 139 [133, 150 mmHg], p = 0.023) but there were no other differences in other cardiovascular risk factors or SLE disease activity (not shown).

**Figure 6:**
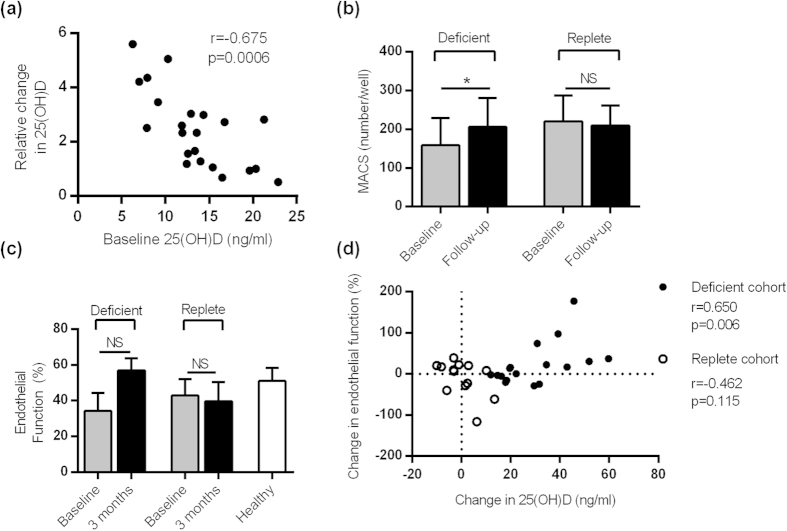
Vitamin D improves endothelial function in SLE patients. (**a**) The change in serum 25(OH)D was strongly inversely correlated with the baseline 25(OH)D (treated patients only). (**b**) The number of MACs significantly increased over 3 months in the treated patients but not the control group. (**c**) There was a non-significant increase in endothelial function (ED/EI) in the treated group but not the control group. The HC group (n = 9) is shown for comparison. (**d**) The change in endothelial function correlated with the change in 25(OH)D in the treated group but not in the replete cohort. The Spearman correlation coefficient for each group is shown. Results show n = 22 deficient and n = 18 replete patients. *P < 0.05, by paired t tests

Compared to vitamin D replete SLE patients, deficient patients showed a significant increase in 25(OH)D following 3 months of cholecalciferol treatment (26.8 [18.3, 35.0] vs. −0.62 [−3.99, 2.63]ng/ml, p < 0.0001). The change in serum 25(OH)D was subject to a threshold effect with a negative correlation between the change in 25(OH)D and the pre-treatment 25(OH)D level (r = −0.675, p < 0.001) (Fig. 6a). Vitamin D treatment had no clinically significant effect on serum calcium (median [IQR] change of 0.06 [−0.05, 0.15] vs. −0.03 [−0.10, 0.03]mmol/l, p = 0.053).

Cholecalciferol increased the number of MACs over 3 months, with an absence of this effect in the control group (Fig. 6b). There was a trend towards improvement in endothelial function in the treated group (ED/EI 47.0% to 62.0%, p = 0.190) but not the replete group (ED/EI 46.2% to 38.5%, p = 0.684) (Fig. 6c). Post-treatment endothelial function was similar to that of a cohort of healthy controls (ED/EI 62.0% vs. 54.2%). There was a significant correlation between the change in 25(OH)D (ΔvitD) and change in endothelial function (ΔEF in the treated group (r = 0.650, p = 0.006) but not the replete group (Fig. 6d). In an ordered logistic regression model, ΔEF was associated with ΔvitD after adjustment for age (OR 1.12 [1.02, 1.24], p = 0.017).

This was a clinically stable population of SLE patients and as such there was no significant change in the SLEDAI score in either group (median change of 0 points in both groups). The changes in endothelial function were therefore independent of SLE disease activity.

## Discussion

Cardiovascular disease remains an important cause of morbidity and mortality both in patients with SLE and other inflammatory diseases. Endothelial repair is a recently recognised phenomenon in which damaged vasculature is restored by circulating populations of cells including MACs. In chronic disease states, defects in the number or function these pro-angiogenic myeloid cells are associated with both prevalent CVD and an increased risk of a cardiovascular event^28^,^29^. Other groups have shown that SLE patients have fewer MACs, which display impaired global function related in part to the presence of increased levels of IFNα^7^,^9^,^30^,^31^.

We have shown that CFUs from SLE patients were both fewer and smaller than in healthy subjects, representing a global impairment of MAC function, supporting the data by Moonen *et al.*^30^. However, this is the first study to show increased MAC numbers in SLE, which may be a compensatory change, as SLE MACs exhibit impaired function in terms of migration and angiogenic capacity. Importantly, this study shows that SLE MACs demonstrate impaired migratory capacity towards SDF-1α *in vitro* which may contribute to the impaired endothelial repair reported in this patient group. This is of interest as Wong *et al.* (2014) reported that calcitriol increased in re-endothelialisation and angiogenesis in murine models of endothelial damage. In these experiments, the homing of MACs to sites of vessel injury was SDF-1α-dependent^25^.

Myeloid cells are known to express functional VDRs and respond to calcitriol *in vitro*^24^. During the early stages of PBMC culture, we demonstrate a transient increase in the expression of the VDR before returning to basal levels, suggesting that MAC phenotype and/or function may be modified by local calcitriol concentrations during the onset of myeloid cell differentiation into MACs. It is not clear whether this change in VDR expression occurs within individual cells (thus changing their sensitivity to vitamin D) or represents a global shift in the myeloid cell phenotype during differentiation. The phenotype of SLE MACs was abnormal but was modified by calcitriol resulting in the normalisation of cell surface markers. Our observation that IL-6 production by MACs was reduced by calcitriol supports mRNA data from PBMCs^26^ and is of potential clinical relevance given the increased risk of CVD mortality associated with raised levels of IL-6^32^. Although the change in surface makers and reduced IL-6 expression suggests that disease activity may be modified by calcitriol, this has not yet been demonstrated. In the randomised controlled trial by Aranow *et al.* (2015), cholecalciferol supplementation did not reduce expression of the IFN signature^33^. In this study, only 16/33 patients receiving vitamin D were replete by the end of the study period which may have significantly reduced the power of the study to detect a change in IFN pathway activation.

The angiogenic capacity of MACs is fundamental to their role in endothelial repair and we identified a number of pro- and anti-angiogenic factors differentially regulated by calcitriol in PBMCs. Amongst them, CXCL-10 (IP-10) has been reported to inhibit angiogenesis in a HUVEC Matrigel model^27^ and *in vivo*^34^. Furthermore, CXCL-10 expression is regulated by IFNα and is expressed in the serum of SLE patients, and further increased during disease flare^35^,^36^. Our data therefore suggests that vitamin D may exert a vasculoprotective role in SLE by reducing MAC secretion of CXCL-10.

We did not investigate the effects of calcitriol in MACs from HCs beyond our IFN model. It remains uncertain if our observations are specific to SLE, or whether they represent a more general effect of calcitriol on MACs. We propose that further studies should determine whether similar observations are seen in healthy subjects and indeed other inflammatory diseases.

The number of MACs has previously been associated with endothelial function in SLE patients^9^. In our study, the number of MACs increased in SLE patients treated with cholecalciferol, but not in vitamin D replete control patients. Although the number of MACs isolated did not directly correlate with endothelial function, the change in ED/EI was strongly correlated with the change in 25(OH)D which may suggest that larger changes in vitamin D levels are needed. One potential mechanism, by which endothelial function may improve, is suggested by our observation that MACs reduced TNF-mediated eNOS down-regulation in endothelial cells and that this effect was augmented by calcitriol. A similar association between improvement in endothelial function *in vivo* and changes in eNOS expression *in vitro* has been demonstrated for other vasculoprotective drugs^37^,^38^.

Our finding that ED/EI improved after cholecalciferol replacement, and correlated with change in 25(OH)D levels, suggests a clinically relevant role for vitamin D on vascular health in SLE. However, in order to study clinically relevant end-points, a formal clinical trial will be necessary. Our data also suggest that a threshold effect exists which warrants further study. Similarly, the dose, duration of effect and ideal target serum level of 25(OH)D for optimal vascular health may not necessarily be the same as those for bone health^39^. A recent pilot study by Kamen and Oates (2015) identified a similar effect of cholecalciferol supplementation on endothelial function in vitamin D deficient SLE patients^40^. Importantly, the authors reported that a target 25(OH)D of ≥32 ng/ml was needed for 50% of patients to show improvement in FMD, supporting the idea of a threshold effect. Finally, it is important to determine whether cholecalciferol treatment has any negative effects on vascular health in SLE. This population has a significant prevalence of CKD and some studies suggest that vitamin D may exacerbate hypercalcaemia and promote vascular calcification in this context^41^.

In conclusion, we have demonstrated that vitamin D can positively modulate endothelial function in patients with stable SLE, independently of disease activity, and that SLE MAC function is augmented by calcitriol in *in vitro* models. These observations support a role for vitamin D in improving cardiovascular health and reducing cardiovascular risk in SLE.

## Methods

### Subject recruitment

Clinically stable SLE patients were recruited from a single centre (Central Manchester University Hospitals) and healthy control subjects from the University of Manchester. Serum 25(OH)D was measured by liquid chromatography-mass spectroscopy (LC-MS). Vitamin D deficiency was defined as 25(OH)D < 20 ng/ml and deficient patients were treated with high dose oral cholecalciferol (typically 400,000IU followed by 20,000IU weekly) according to regional guidelines under the supervision of their usual physician in an observational manner. Vitamin D replete (<30 ng/ml) were not treated and acted as a control cohort. SLE disease activity was measured using the Systemic Lupus Erythematosus Disease Activity Index 2000 (SLEDAI-2 K) scoring system.

### Measurement of endothelial function

Endothelial function was measured by a single operator using flow-mediated dilatation (FMD) as described previously^42^. Briefly, subjects were fasted overnight and rested in a temperature-controlled room. A blood pressure cuff was placed on the forearm and inflated to supra-systolic pressure for 5 minutes. The brachial artery was visualised using a Philips iU-22 ultrasound machine and the diameter was measured off-line on a frame-by-frame basis for a period of 2 minutes. The mean maximal diameter was calculated using a cubic spline curve. FMD was defined as the % change in diameter between baseline and mean maximal diameter. Dilatation was also measured in response to sublingual glyceryl trinitrate (GTN). Endothelial function was expressed as the ratio of endothelial-dependent (FMD) to endothelium-independent (GTN-mediated) dilatation (ED/EI) in order to correct for differences in baseline arterial diameter and subject age.

### MAC isolation from whole blood

MACs were isolated by density separation of 50 ml whole blood in EDTA on Ficoll-paque PLUS (GE Healthcare Life Sciences, UK). PBMCs were cultured at a density of 1 × 10^6^ cells/cm^2^ in endothelial cell media with 20% FCS (EGM, Lonza, Switzerland) on human fibronectin for 7 days. Culture media was changed on day 4. The MAC phenotype was confirmed by Dil-Ac-LDL uptake and UEA-1 lectin binding as described previously^7^. For functional assays MACs were used after 7 days and conditioned media obtained at the same time point. SLE MACs were also cultured in the presence of 1, 25(OH)_2_D_3_ (MAC-D).

### Colony Forming Unit (CFU) assay

The CFU assay was performed in a separate SLE and healthy control cohort and was adapted from that reported by others^29^,^43^ PBMCs were isolated from 20 ml EDTA blood as described above. PBMCs were suspended in EGM-2 (with 20% FCS (Lonza, Switzerland) and plated at 2 × 10^6^ cells/cm^2^ onto human fibronectin-coated wells. After 48 hours, non-adherent cells were removed, washed, and re-plated onto fibronectin. Cultures were maintained for a further 5 days. Colonies were enumerated manually and defined as a group of central rounded cells with peripheral spindle-shaped cells.

### Adhesion assay

MAC adhesion to a TNFα-activated endothelial monolayer was measured. MACs were detached and labelled using Cell Tracker™ Green CMFDA (Molecular Probes, USA). Confluent monolayers of human aortic endothelial cells (HAoEC, Promocell, Germany) were treated for 6 hours with 10 ng/ml TNFα. We have previously demonstrated that this is sufficient to up-regulate expression of adhesion molecules in this cell type. MACs were allowed to adhere to the HAoECs for 1 hour, non-adherent cells removed, and the number of MACs quantified in random fields. Relative adhesion was expressed and the mean number of cells adherent to TNFα-activated HAoECs compared to resting endothelial cells.

### Migration assay

The migration of MACs towards SDF-1α was determined by Transwell® migration assays. MAC and MAC-D were serum and growth factor-starved for 3 hours and then detached and re-suspended in basal EBM +1% (w/v) bovine serum albumin. 1 × 10^6^ cells were added to each fibronectin-coated Transwell® chamber (8 μm pore size) and allowed to migrate towards 100 ng/ml SDF-1α or EBM alone for 8 hours. The upper surface of the membrane was scraped clean and inserts were fixed in 100% ice-cold methanol overnight. Migrated MACs were stained with Mayer’s Heamulum for 5 minutes, mounted onto glass slides and the number of MACs was enumerated in 3 random fields per insert. The relative migration was expressed as the number of migrated cells in chambers containing SDF-1 compared to EBM alone.

### Matrigel™ network formation assay

Network formation assays were used to determine the angiogenic capacity of MAC and MAC-D. 3.5 × 10^3^ HAoECs were plated onto growth factor-reduced Matrigel™ in Ibidi μ-angiogenesis slides, in MAC or MAC-D conditioned media. Endothelial media (EGM) (±10 nM 1, 25(OH)_2_D_3_) was used as a control. HAoEC networks were allowed to form for 14 hours and a single image obtained from each well. Networks were quantified in terms of the number of closed loops and the total pixel area (see supplementary methods).

### Real-time PCR analysis

RT-PCR was performed using RNA obtained from PBMCs, MACs and HAoECs. RNA was isolated from PBMCs, MAC/MAC-D and HAoECs using the phenol-cholorform method with DNase 1 (Ambion, UK) to remove genomic DNA. 1 μg RNA was reverse transcribed using the Precision nanoscript™ Reverse transcription kit (PrimerDesign Ltd, UK) according to the manufacturer’s protocol. Real-time qPCR was conducted using the SYBR green method on a 7500 Fast Real-Time PCR System (Applied Biosystems, UK). All PCR reactions were conducted using the Precision™ Mastermix with low ROX (PrimerDesign Ltd, UK). All primers were obtained from PrimerDesign (see supplementary Table). Gene expression was normalised to the geometric mean of CYC1 and ATP5B using the 2-ΔCt calculation.

### Immunoblotting

Protein lysates from MACs and HAoECs were resolved on SDS-PAGE and transferred to nitrocellulose for blotting. In MACs antibodies against VDR (sc-13133, Santa Cruz, USA) and GAPDH (5174, Cell Signalling USA) were used. In HAoECs antibodies against eNOS (Cell Signalling, USA) and α-tubulin (Cell Signalling, USA) were used. HRP-conjugated secondary antibodies were obtained from Jackson Immunoresearch, USA.

### MAC expression of IL-6 and CXCL-10

Cytokines in MAC-conditioned media were measured by colorimetric ELISA using the IL-6 Human ELISA kit (Abcam, UK) and Human CXCL-10/IP10 ELISA kit (Invitrogen, USA) respectively, according to the manufacturer’s instructions.

### Statistical methods

Patient-level data were compared using the Mann-Whitney U test (and Spearman’s correlation), whilst data from *in vitro* models were compared using either a two-tailed t test or paired t test as appropriate. The laboratory data is presented as mean ± SEM (unless described otherwise) of n ≥ 3 independent experiments. *P* < 0.05 was considered to be statistically significant.

### Study approval

The study was approved by North West 1 Research Ethics Committee (11/NW/0008). All study participants provided written informed consent. The study conforms to the tenets of the Declaration of Helsinki.

## Additional Information

**How to cite this article**: Reynolds, J. A. *et al.* Vitamin D improves endothelial dysfunction and restores myeloid angiogenic cell function via reduced CXCL-10 expression in systemic lupus erythematosus. *Sci. Rep.* **6**, 22341; doi: 10.1038/srep22341 (2016).

## Supplementary Material

Supplementary Information

## Figures and Tables

**Table 1:** Baseline characteristics of the vitamin D deficient and replete groups.

Demographics	Deficient (n = 22)	Replete (n = 18)	p
Age (years)	46.9 (44.5, 57.6)	57.8 (52.7, 64.6)	0.0066
Disease duration (years)	12.7 (6.94, 18.7)	25.1 (10.6, 31.4)	0.116
Caucasian	16 (72.7%)	17 (94.4%)	0.072
Ethnic Origin			0.406
Caucasian	16	17	
Black Caribbean	2	1	
Pakistani	2	0	
Chinese	1	0	
Other	1	0	
Waist circumference (cm)	83.5 (71.0, 91.0)	85.5 (76.0, 95.0)	0.596
Hip circumference (cm)	98.5 (88.0, 105)	93.0 (89.5, 104)	0.913
BMI (kg/m^2^)	25.4 (22.0, 29.1)	25.0 (21.3, 28.8)	0.568
Vitamin D Status			
25(OH)D (ng/ml)	13.1 (10.3, 16.5)	34.5 (30.8, 40.2)	<0.001
PTH (pg/ml)	47.0 (38.0, 66.0)	37.0 (29.0, 50.0)	0.096
Calcium	2.34 (2.24, 2.38)	2.36 (2.32, 2.42)	0.634
Vitamin D supplements			0.005
None	14	6	
Low dose	8	5	
High dose	0	7	
Risk Factors for Vitamin D Deficiency			
Self-reported photosensitivity	15 (68.2%)	7 (38.9%)	0.064
Active sun avoidance	14 (63.6%)	10 (55.6%)	0.604
High-factor sunblock use	12 (54.5%)	8 (44.4%)	0.525
Covered arms/legs	9 (40.9%)	1 (5.60%)	0.010
Covered face	0	0	—
Malabsorption syndrome	0	0	—
SLE Disease Features			
No. ACR criteria	5 (4, 6)	5 (4, 6)	0.748
Renal disease (ever)	7 (31.8%)	1 (5.60%)	0.039
ANA positive (ever)	21 (95.4%)	16 (88.9%)	0.433
Steroids OR immunosuppressant	13 (59.1%)	3 (16.7%)	0.006

Comparisons between the groups were made using Mann-Whitey U-tests for continuous data and the Chi-squared test for categorical data. *P* < 0.05 was considered to be statistically significant.
