# Supplementary material for: Vitamin D improves endothelial dysfunction and restores myeloid angiogenic cell function via reduced CXCL-10 expression in systemic lupus erythematosus
Source: Sci Rep. 2016 Mar 1;6:22341. doi: 10.1038/srep22341 (PMC4772382; doi:10.1038/srep22341)
Supplement: Supplementary Information [file srep22341-s1.doc]

**Vitamin D improves endothelial dysfunction and restores myeloid angiogenic cell function via reduced CXCL-10 expression in systemic lupus erythematosus**

John A Reynolds, Sahena Haque, Kate Williamson, David W Ray, M Yvonne Alexander, Ian N Bruce

**SUPPLEMENTARY METHODS AND DATA**

**SUPPLEMENTARY METHODS**

**Interaction between MACS and HAoECs**

To investigate the *in vitro* interaction between endothelial cells and MACs, MACs were labelled with MACs were detached and labelled using Cell Tracker™ Green CMFDA (Molecular Probes, USA), and co-cultured with HAoECs for 14 hours on Matrigel. Time-lapse microscopy was used over 14 hours to track MAC migration along with HAoECs forming tubules and toward established HAoEC networks. Images were collected on a Olympus BX51 upright microscope using a 10x objective and captured using a Coolsnap ES camera (Photometrics) through MetaVue Software (Molecular Devices).

**RT-qPCR**

All primer pairs were obtained from PrimerDesign (Southampton, UK) and optimised for RT-qPCR. The sequences for the reference genes ATP5B and CYC1 (used in both MACs and HAoECs) are not available for publication

**Supplementary Table S1:**

|  | Primer sequences (5’-3’) | |
| --- | --- | --- |
|  | Forward | Reverse |
| MAC Primers |  | |
| CD14 | CCTAAGATCCAAGACAGAATAATGAA | TTTTAATAAAGGTGGGGCAAAGG |
| CD68 | ACCACCACTCACAGGACAA | GTGCTATTGCTTGTTGGATGAA |
| CD86 | TTGATAATGGGATGAATGGAAGGA | CGTAGGACATCTGTAGGCTAAG |
| CD206 | TGGGTTCCTCTCTGGTTTCC | CAACATTTCTGAACAATCCTATCCA |
| CCR7 | AAGCCTGGTTCCTCCCTATC | ATGGTCTTGAGCCTCTTGAAATA |
| HAoEC Primers |  | |
| eNOS | ACAAGAGTTATAAGATCCGCTTCAA | CCTGCACTGTCTGTGTTACTG |

**Analysis of tubule network density**

An algorithm was developed to allow the quantification of HAoEC tubule network parameters modified from that described by Guidolin *et. al*. (2010). Images of the networks were imported into ImageJ software and the network reduced to a single pixel thickness. The original image underwent background subtraction and was transformed into a binary image. The gaps between cells were closed and the image converted to a skeleton image with branches a single pixel in diameter. The “Analyse Skeleton” plug-in for ImageJ was then used to quantify parameters of the network in terms of the number of branches, number of junctions and total pixel area. The number of closed loops/polygons was enumerated manually {Arganda-Carreras, 2010 762 /id}.

**Pro-angiogenic factor secretion by MACs**

The secretion of angiogenic factors by MACs was measured using a Bio-Plex Pro™ Human Angiogenesis 9-Plex Panel suspension array according to the manufacturer’s instructions. Samples were thawed on ice and standards diluted in EGM 20 FCS%. The concentration of angiogenic factor was extrapolated from the standard curve and reported as pg/ml.

**Identification of novel angiogenic factors**

In the study by Kupfer *et. al.* (2013) transcriptional profiling on PBMCs from 12 healthy subjects in the presence or absence of 10nM 1,25(OH)2D3 for 24 hours was undertaken using an Illumina HumanHT-12 v3 Expression Array {Kupfer, 2013 1138 /id}. The normalised dataset is freely available in the Gene Expression Omnibus (GEO) (http://www.ncbi.nlm.nih.gov/geo; accession number GSE50012). Samples were analysed using GEO2R software to identify transcripts which were differentially expressed between PBMCs treated with 1,25(OH)_2_D_3_ and vehicle (ethanol). The resultant dataset was explored using Ingenuity IPA Software (Qiagen, Redwood City, USA). Differentially regulated genes were defined as those with a log-fold change of 0.39 with p≤0.01. This approach was used in the investigation of phenotypic changes in response to calcitriol and in the determination of differentially regulated angiogenic factors.

**SUPPLEMENTARY RESULTS**

**MACs interact with endothelial cells and promote angiogenesis**

First, to examine the interaction between MACs and endothelial cells, HC MACs and HAoECs were co-cultured on Matrigel™. HAoECs alone formed complex branching networks, whilst MACs alone did not. Co-culture demonstrated that MACs co-localised with HAoECs networks (figure S1A). Next, to determine whether MACs truly migrated towards HAoECs, we added MACs to existing HAoEC networks. MACs migrated towards and adhered to HAoECs over 5 hours (figure S1B). In order to quantify this effect, the migration of HC MACs towards SDF-1α was examined over 8 hours in modified Boyden chambers. MACs displayed chemotaxis towards SDF-1α over this time period (figure S1C) and were also able to adhere preferentially to an activated endothelial cell monolayer. After 1 hour, the number of MACs adhering to TNFα-treated HAoECs was greater than control HAoECs (figure S1D).

The angiogenic capacity of HC MACs was quantified using an *in vitro* Matrigel™ network formation assay. HAoEC network density was increased in HAoECs suspended in MAC-conditioned media compared to endothelial growth media alone (figure S1E). Quantification of the density showed that MAC-conditioned media significantly increased the network complexity in terms of the number of closed loops (figure S1F) and the total network area (figure S1G).

**Figure S1**


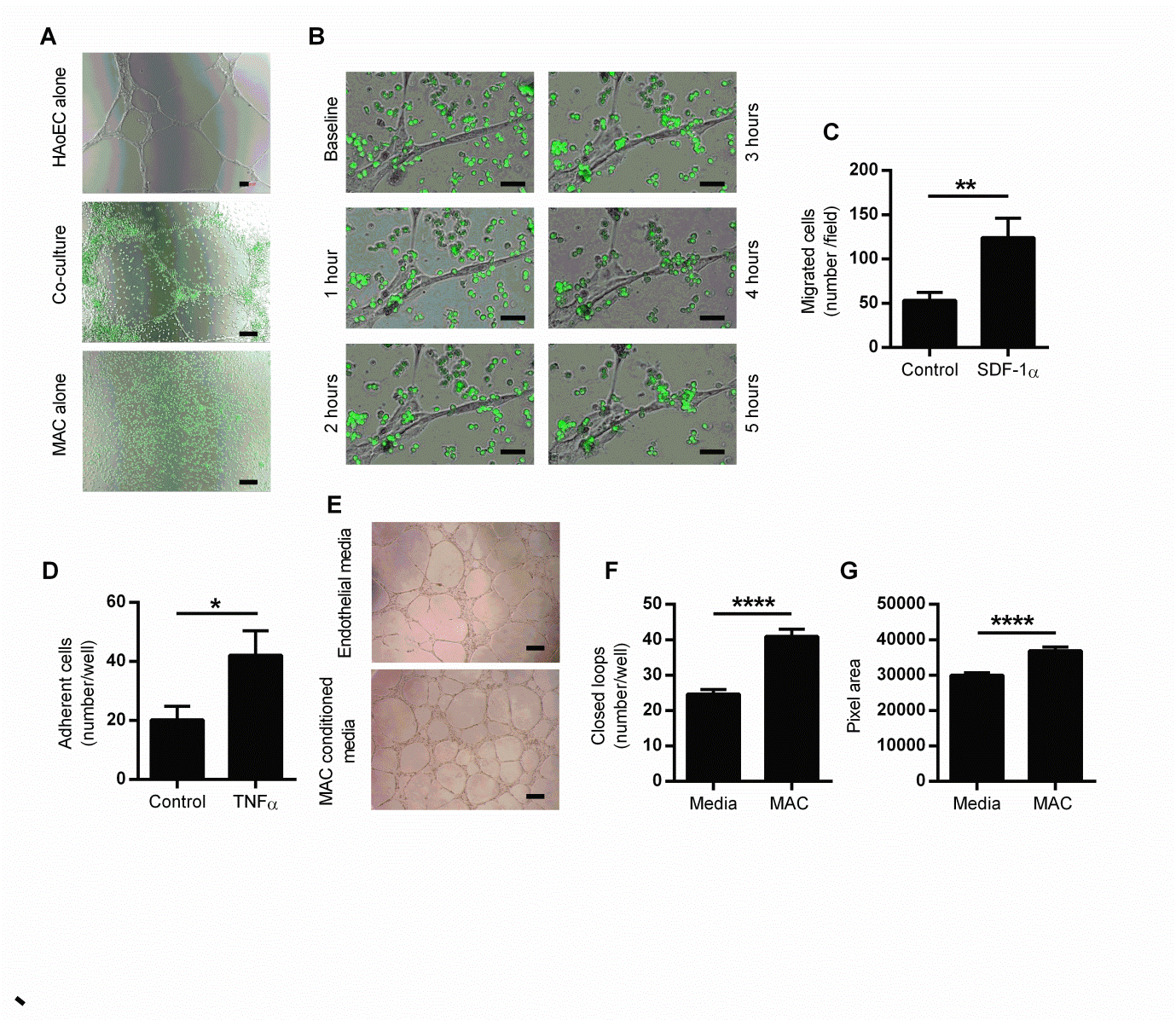


(A) Healthy MACs align to HAoECs networks on Matrigel but are unable to form networks alone. MACs appear green. Scale bars: 100µm. (B) Time-lapse microscopy to show the migration of MACs (green) towards a HAoEC network. Scale bars: 20µm. (C) Transwell migration assays as described in Methods show that SDF-1α acts as a chemotactic factor for MACs (n=6 healthy subjects). (D) MACs preferentially adhere to activated rather than resting endothelial cells (n=4 healthy subjects). (E) The conditioned media from healthy MACs increases the density of the network formed by HAoECs compared to the media alone. Scale bars: 250µm. (F) MAC conditioned media significantly increases the density of the network in terms of the number of closed loops in the HAoECs network compared to media alone. (G) The size of the HAoEC network is increased by MAC conditioned media. Data in C and D show the mean ± SE MACs in 3 random fields per subject in duplicate and triplicate respectively. Data in F and G use MAC conditioned media generated from 9 individuals. **P*<0.05, ***P*<0.01, *****P*<0.0001 by ratio paired t-tests.

**Pro-angiogenic factor secretion by MACs**

The angiogenic profile of both HC and SLE MACs was studied using a multiplex assay. The Bio-Plex Pro Human Angiogenesis Assay (Bio-Rad Ltd, UK) quantifies 9 common angiogenic factors: Vascular endothelial growth factor-A (VEGF-A), platelet-derived growth factor-BB (PDGF-BB), platelet endothelial cell adhesion molecule-1 (PECAM-1), interleukin-8 (IL-8), hepatocyte growth factor (HGF), leptin, follistatin, angiopoeitin 2 and granulocyte-colony stimulating factor (G-CSF). These factors were measured in both HC MAC-conditioned media and in EGM-20% FCS alone (to exclude contribution of factors in the FBS or bovine brain extract within the media).

None of the 9 factors in the assay were added to EGM as a recombinant growth factor. Of the 9 factors measured, all were detected in the conditioned media with the exception of angiopoetin-2. The conditioned media contained significantly higher concentrations of VEGF-A, PDGF-BB, PECAM-1 and HGF with a non-significant trend towards higher concentration of IL-8, leptin and follistatin (figure S2). There were only very low concentrations of G-CSF in conditioned media which was not significantly different from EGM alone.

The expression profiles were then compared using conditioned media (at day 8) from untreated and vitamin D-treated lupus MACs and untreated and IFNα-treated HC MACs. A low concentration (0.1ng/ml) IFNα2b was used to module the MAC response without causing significant reduction in cell number. There was no significant difference in the expression of any of these factors between HC and SLE MACs (figure 6-18). In lupus MACs there was little change in the secretion of pro-angiogenic factors in response to 10nM 1,25(OH)2D3. The exception was a significant reduction in follistatin from a mean (sd) 42.9 (25.7) to 21.7 (12.9) pg/ml (p=0.044). In contrast, there was no difference in follistatin concentration between HC and IFN-treated HC conditioned media (29.6 [16.0] vs. [28.8 [14.4] pg/ml, p=0.905).

Whilst 1,25(OH)2D3 had only a modest effect on lupus MACs, IFNα resulted in significant changes in the expression of 4 angiogenic factors by HC MACs. There was a significant reduction in the concentration of HGF when MACs were cultured with IFN (1584 [664] vs. 646.6 [269.4] pg/ml, p=0.0017). The other factors which were changed by IFN were all increased: VEGF-A (p=0.016), leptin (p=0.049) and IL-8 (p=0.021).

**Figure S2:**

**
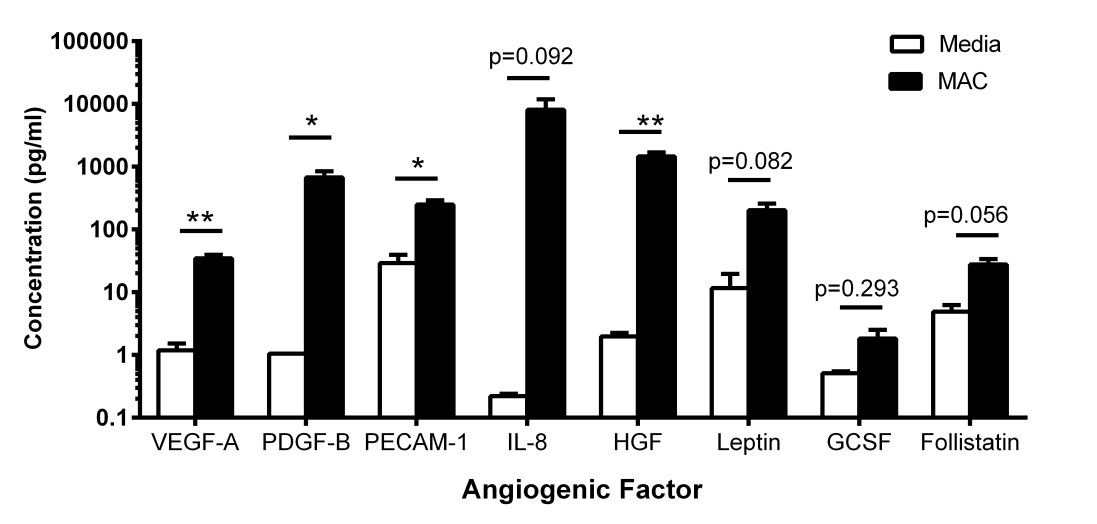
**

The expression of pro-angiogenic factors was measured in the media of MACs and compared to EGM-20% FCS. Angiopoetin-2 was not detectable in any sample (data not shown). MACs secreted significantly higher levels of VEGF-A, PDGF-B, PECAM-1 and HGF, with a trend towards increased IL-8, leptin and follistatin. The columns show the mean concentration in pg/ml on a logarithmic scale and the bars show standard error. A total of n=3 growth media samples and n=8 healthy MAC samples were analysed. Comparisons were made by t test, *p<0.05, **p<0.01.

**Figure S3:**

**
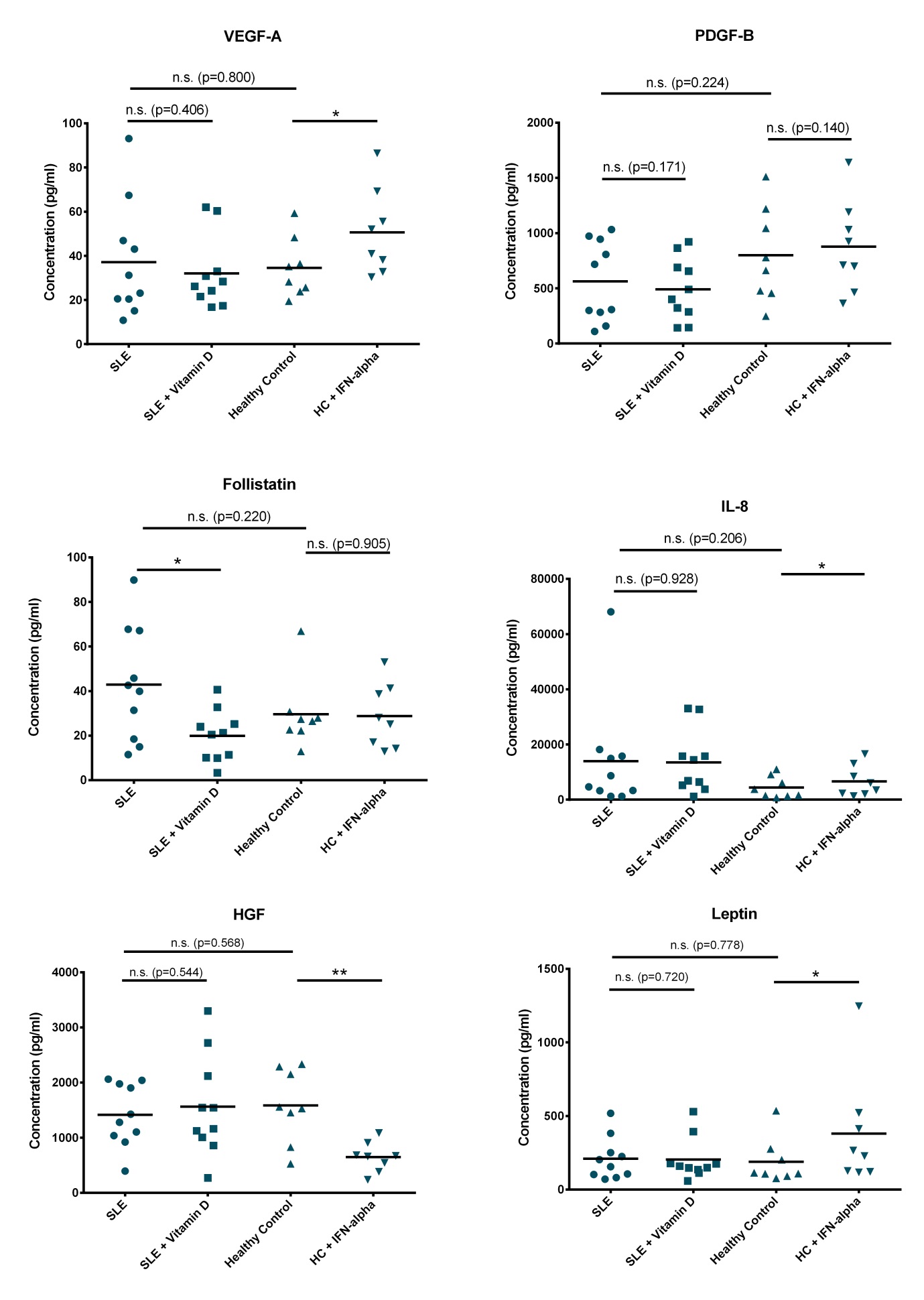
**

The concentration of pro-angiogenic factors in the media of SLE and HC MACs cultured in the presence/absence of 10nM 1,25(OH)2D3 or 0.1ng/ml IFNα2b was measured using a multiplex array. Paired samples were measured from MACs derived from n=10 SLE patients and n=8 healthy controls. HC MACs treated with IFNα expressed significantly lower levels of HGF and increased levels of IL-8, leptin and VEGF-A. Vitamin D resulted in a statistically significant reduction in the expression of follistatin. No differences were seen between any of the conditions and levels of PECAM-1 or GCSF (data not shown). The graph shows the mean values for each cytokine. Comparisons were made by paired (SLE MAC ± vitamin D, and HC ± IFNα) or unpaired (SLE MAC vs. HC MAC) t tests, *p<0.05, **p<0.01.

**Identification of novel angiogenic factors regulated by calcitriol**

The dataset from Kupfer *et. al.* (2013) described above was used to identify which secrete pro-angiogenic factors were up-regulated by 1,25(OH)2D3 and which anti-angiogenic factors were down-regulated in PBMCs after 24 hours. The factors up- and down-regulated are shown in tables S2 and S3 respectively.

Table S2:

| Pro-angiogenic factors up-regulated | | | | |
| --- | --- | --- | --- | --- |
| Gene Symbol | Name | Log Fold Change | P value | Effect |
| LAMC1 | laminin, gamma 1 | 1.20 | 2.49x10^-7^ | Increases tubule formation by endothelial cells – important component of Matrigel |
| PRL | prolactin | 1.04 | 0.0019 | Recruit HUVECs and stimulate angiogenesis in tumour cells |
| HTRA1 | HtrA serine peptidase 1 | 2.27 | 1.29x10^-4^ | Regulation of retinal angiogenesis – cleaves IGF-binding proteins |
| ORM1 | orosomucoid 1 | 2.76 | 1.01x10^-6^ | Stimulates angiogenesis in the CAM assay |
| OSM | oncostatin M | 2.72 | 7.88x10^-12^ | Induces angiogenesis in vitro and in vivo – increased endothelial proliferation, increased migration, increased tubule formation of dermal microvascular cells but NOT HUVEC. |
| GREM1 | gremlin 1, DAN family BMP antagonist | 0.65 | 2.41x10^-4^ | Novel agonist at VEGFR2 |
| CXCL12 | chemokine (C-X-C motif) ligand 12 (SDF-1) | 0.59 | 1.1x10^-5^ | Stimulates cell proliferation and capillary tube formation, attenuates HMEC apoptosis. |
| PLAU | plasminogen activator, urokinase (uPA) | 0.739 | 4.12x10^-4^ | Higher endothelial levels of uPA correlate with increased angiogenic capacity. |

Table S3:

| Anti-angiogenic factors down-regulated | | | | |
| --- | --- | --- | --- | --- |
| Gene Symbol | Name | Log Fold Change | P value | Effect |
| APOE | Apolipoprotein E | -0.7 | 4.72 x 10^-4^ | Binds to HSPGs on cell surface, blocking binding of growth factors e.g. VEGF |
| THBS1 | Thrombospondin 1 | -2.62 | 2x10^-4^ | Inhibits endothelial cells adhesion, motility and growth. It also interacts with numerous proteases involved in angiogenesis, including plasminogen, urokinase, matrix metalloproteinase, thrombin, cathepsin, and elastase |
| CXCL9 | Chemokine (C-X-C motif) ligand 9 | -2.9 | 7.7310^-6^ | Reduces VEGFR2 (KDR), phospholipase Cγ (PLCγ), and extracellular signal-regulated kinase (ERK) phosphorylation - a direct counter-regulatory molecule of VEGF signalling |
| GSN | Gelsolin | -1.56 | 1.53x10^-10^ | Regulator of actin cross linkage – roll in angiogenesis not clear |
| MMP12 | matrix metallopeptidase 12 (macrophage elastase) | -0.54 | 0.00732 | MMP-12 is a key regulator of macrophage infiltration and inflammation, contributing to retinal vascular dysfunction and pathological angiogenesis  MMP-12 may block angiogenesis by converting plasminogen to angiostatin, which is one of the most potent angiogenesis antagonists. |
| VASH1 | vasohibin 1 | -0.715 | 1.11x10^-7^ | Inhibits migration, proliferation and network formation by endothelial cells as well as angiogenesis. This inhibitory effect is selective to endothelial cells as it does not affect the migration of smooth muscle cells or fibroblasts. |
| CXCL10 | chemokine (C-X-C motif) ligand 10, IP10 | -2.88 | 3.85x10^-8^ | Inhibits endothelial cell proliferation. Inhibits basic fibroblast growth factor-induced neovascularization of Matrigel injected subcutaneously into athymic mice. In addition suppresses endothelial cell differentiation into tubular capillary structures *in vitro*. No effect on endothelial cell growth, attachment, and migration as assayed *in vitro*. |
| CCL13 | chemokine (C-C motif) ligand 13 | -3.71 | 2.86x10^-13^ | Important for leucocyte recruitment – role in angiogenesis not clear |

Reference:

Guidolin,D., Albertin,G., and Ribatti,D. 2010. Exploring in vitro angiogenesis by image analysis and mathematical modeling. In *Microscopy: Science, Technology, Applications and Education, Volume 2*. A.Mendez-Vilas, and Badajoz,D., editors. Formatex. Spain. 876-884.
